# What Next?

**Published:** 1856-01

**Authors:** 


*What Next?*—We see it stated in the papers that Mr. Charles Goodyear, Jun., now a resident of Paris, has recently obtained letters patent for constructing bases of artificial teeth consisting of a hard compound of caoutchouc and gutta percha mixed with sulphur and then subjected to heat, forming a vulcanized compound of the two first articles. We presume the caoutchouc and gutta percha are first bleached white, and afterwards made of a gum-color by mixing with them suitable coloring matter. Whether the compound will resist for a length of time the action of the secretions of the mouth we are not informed. If it will do this, it may prove a valuable acquisition to the resources of the dental art, provided artificial teeth can be as lightly and securely mounted on a base of this sort as on a metallic plate. These are questions which we have not yet seen answered.

